# Short-term outcome of 1,465 computer-navigated primary total knee replacements 2005–2008: A report from the Norwegian Arthroplasty Register

**DOI:** 10.3109/17453674.2011.575743

**Published:** 2011-07-08

**Authors:** Øystein Gøthesen, Birgitte Espehaug, Leif Havelin, Gunnar Petursson, Ove Furnes

**Affiliations:** ^1^Haugesund Hospital, Haugesund; ^2^The Norwegian Arthroplasty Register, Department of Orthopaedic Surgery, Haukeland University Hospital, Bergen; ^3^Department of Surgical Sciences, Institute of Medicine and Dentistry, University of Bergen, Bergen; ^4^Lovisenberg Diakonale Hospital, Oslo, Norway

## Abstract

**Background and purpose:**

Improvement of positioning and alignment by the use of computer-assisted surgery (CAS) might improve longevity and function in total knee replacements, but there is little evidence. In this study, we evaluated the short-term results of computer-navigated knee replacements based on data from the Norwegian Arthroplasty Register.

**Patients and methods:**

Primary total knee replacements without patella resurfacing, reported to the Norwegian Arthroplasty Register during the years 2005–2008, were evaluated. The 5 most common implants and the 3 most common navigation systems were selected. Cemented, uncemented, and hybrid knees were included. With the risk of revision for any cause as the primary endpoint and intraoperative complications and operating time as secondary outcomes, 1,465 computer-navigated knee replacements (CAS) and 8,214 conventionally operated knee replacements (CON) were compared. Kaplan-Meier survival analysis and Cox regression analysis with adjustment for age, sex, prosthesis brand, fixation method, previous knee surgery, preoperative diagnosis, and ASA category were used.

**Results:**

Kaplan-Meier estimated survival at 2 years was 98% (95% CI: 97.5–98.3) in the CON group and 96% (95% CI: 95.0–97.8) in the CAS group. The adjusted Cox regression analysis showed a higher risk of revision in the CAS group (RR = 1.7, 95% CI: 1.1–2.5; p = 0.02). The LCS Complete knee had a higher risk of revision with CAS than with CON (RR = 2.1, 95% CI: 1.3–3.4; p = 0.004)). The differences were not statistically significant for the other prosthesis brands. Mean operating time was 15 min longer in the CAS group.

**Interpretation:**

With the introduction of computer-navigated knee replacement surgery in Norway, the short-term risk of revision has increased for computer-navigated replacement with the LCS Complete. The mechanisms of failure of these implantations should be explored in greater depth, and in this study we have not been able to draw conclusions regarding causation.

The role of computer navigation in knee replacement surgery is still under debate (Bauwens et al. 2007, Kim et al. 2009, Longstaff et al. 2009). Improvement of positioning and alignment by using computer navigation might also improve longevity and function, but there is little evidence. The high costs of computer navigation equipment are inclined to make any improvement less cost-effective (Slover et al. 2008).

Increased costs, the time-consuming nature of the method, and a possible new source of complications—i.e. fractures and infection—are some of the arguments against using computer navigation. In Norway, 11% of the knee replacements performed during 2005–2008 were reported to be implanted using computer navigation (Furnes et al. 2008).

We evaluated the short-term results of computer-navigated primary total knee replacements (CAS) without patella resurfacing, by comparing them to the results of conventionally operated total knee replacements (CON) performed using alignment guides. Revision for any reason was the primary outcome. Intraoperative complications, causes of revision, and operating time were secondary outcomes.

## Patients and methods

Primary knee replacements reported to the Norwegian Arthroplasty Register during the period 2005–2008 were included in this prospective observational study. The register was established in 1987 as a hip replacement register (Havelin et al. 2000). The registration of knee replacements started in 1994 (Furnes et al. 2002), but the use of computer navigation was not registered until 2005. At the time of surgery, a form is completed and sent to the register—including information on age, sex, laterality, ASA category, date of surgery, preoperative diagnosis, previous knee surgery, prosthesis type and brand, prophylactic antibiotics, antithrombotic medication, approach (minimally invasive or not), surgical method (use of computer navigation or not, and the name of the system being used), fixation method, intraoperative complications, status of the cruciate ligaments, and whether the present operation was a primary or secondary (revision) procedure. Revision is defined as a complete or partial removal/exchange of the implant, or insertion of a component (including patella button). Primary operations were linked to subsequent revisions by the unique identification number of all Norwegian residents. Of all knee replacements performed in Norway, 99% of all primary operations and 97% of all revisions are estimated to be reported to the register (Espehaug et al. 2006).

### Selection of patients

**Figure 1.**
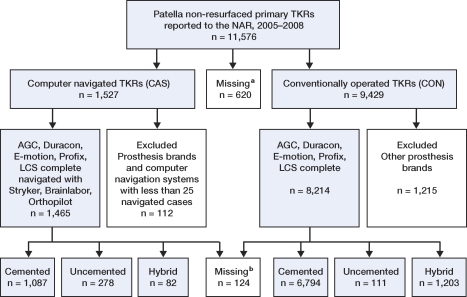
Selection of cases. NAR: Norwegian Arthroplasty Register; TKR: total knee replacement; CAS: computer-assisted surgery (abbreviation for computer-navigated knee replacements in the article), CON: conventionally operated knee replacement, using either intra-medullary or extra-medullary alignment rods.

11,576 non-patella resurfaced primary total knee replacements implanted during the years 2005–2008 were split into 2 groups: CAS and CON (Figure 1). Patella resurfaced knee replacements were excluded from the material due to low numbers (9 in the CAS group and 241 in the CON group).

**^a^** No information on operative technique.
**^b^** No information on fixation method.

In the CAS group, 1,527 operations were performed in 25 orthopedic centers. The number of patients operated with CAS varied from 497 cases reported from 1 center to less than 10 cases, reported from each of 7 centers. 4 computer navigation systems (Brainlab, Orthopilot, Aculumen, and Stryker) and 10 different implants with cemented, uncemented, and hybrid fixation were reported. Only 19 knees were computer-navigated with the use of Aculumen, and they were excluded due to the small number. We selected the 3 most frequently used navigation systems (Brainlab, Orthopilot, and Stryker), along with the 5 most frequently used computer-navigated implants (AGC: Biomet; Duracon: Stryker; e.motion: Aesculap, LCS Complete: DePuy; and Profix: Smith and Nephew) (Figure 1). Implants inserted with a computer-navigated system less than 25 times were excluded, leaving 1,465 computer-navigated knees that were suitable for evaluation.

In the CON group, 9,429 implantations were performed during this time period. From these implantations, only the same prosthesis brands as in the CAS group were selected, giving 8,214 CON knee replacements for comparison.

### Demographics

In the CAS group, there were more males and they were 1 year younger on average than in the CON group (Table 1). Intraoperatively verified deficiency of the ACL, previous surgery of the knee, and the use of uncemented implants were more frequent in the CAS group.

**Table 1.** Demographic data of primary total knee replacements without patella component (computer navigated (CAS) or conventionally operated (CON)) reported to the Norwegian Arthroplasty register, 2005–2008

	CAS	CON	p-value
Number	1,465	8,214	
Men %	39	33	< 0.001
Age, years	68.8	69.8	0.001
95% CI	68.3–69.3	69.5–70.0	0.001
Right knee, %	56	55	0.3
MIS **^a^**, % (n)	0 (7)	0 (21)	< 0.001
ASA category **^b^**, % (n)			0.03
1	19 (272)	22 (1,766)	
2	62 (907)	57 (4,706)	
3	18 (266)	20 (1,630)	
4	0 (2)	0 (14)	
missing	1 (18)	1 (98)	
Diagnosis preoperatively, %			
Primary gonarthritis	90	89	0.1
Other	10	11	
Fixation method, % (n)			< 0.001
Cemented	75 (1,087)	84 (6,794)	
Uncemented	19 (278)	1 (111)	
Hybrid (uncemented femur)	6 (82)	15 (1,203)	
Prosthesis brand, % (n)			< 0.001
AGC	5 (80)	13 (1,072)	
Duracon	11 (168)	5 (443)	
e.motion	21 (300)	0 (7)	
LCS complete	39 (570)	35 (2,834)	
Profix	24 (347)	47 (3,858)	
Prosthesis design, % (n)			
Fixed bearing	41 (595)	65 (5,373)	< 0.001
Mobile bearing	59 (870)	35 (2,841)	< 0.001
Stabilized **^c^**	2 (25)	2 (174)	0.2
Previous operations of the knee, %	37	27	< 0.001
Osteosynthesis affecting the knee joint	3	2	0.02
Osteotomy	5	4	0.3
Synovectomy	2	2	0.9
Other	30	21	< 0.001
Intact ACL**^d^** preoperatively, %	71	81	< 0.001

**^a^** MIS: minimally invasive surgery.

**^b^** ASA category: American Society of Anesthesiologists physical status classification system.

**^c^** Polyethylene insert posteriorly stabilized or other stabilization.

**^d^** ACL: anterior cruciate ligament.

### Statistics

Descriptive analyses were performed to assess baseline characteristics of the study groups. Differences were evaluated using the chi-square test for proportions and the independent-samples t-test for mean values.

The CON group was compared to the CAS group regarding survivorship. Revision for any reason—and secondly, revision due to specific causes—was used as endpoint. Information on deaths or emigrations was retrieved from the National Population Register until December 31, 2009. The survival times of unrevised implants were censored at the last date of observation, meaning the date of death or emigration, or December 31, 2009. Median follow-up was calculated following the reverse Kaplan-Meier method (Schemper and Smith 1996). The Kaplan-Meier method provided unadjusted estimates of survivorship after 1 and 2 years of follow-up. The Cox multiple regression model was used to calculate hazard rate ratios (RRs) for evaluation of the effect of computer navigation on survivorship, with adjustment for potential confounding by age (continuous), sex, ASA category (I, II, III/IV), method of fixation (cemented, uncemented, or hybrid cementation (uncemented femur, cemented tibia)), prosthesis brand, preoperative diagnosis (osteoarthritis, other diagnoses), and previous knee surgery (yes/no). The adjusted RR estimates are presented with 95% confidence intervals (CIs) and p-values relative to the CON group. Additional adjustment for operating time did not alter the RR estimates. Cox regression with use of computer navigation as stratification factor was used to construct survival curves for the treatment groups, with adjustment for the factors described above. Survival curves for the various prosthesis brands were constructed in the same way. In subanalyses, results of computer-navigated and conventionally operated knees were obtained for each prosthesis brand and also according to fixation method (cemented knee replacements, uncemented knee replacements, and hybrid knee replacements).

The proportional hazards assumption of the Cox model was tested based on scaled Schoenfeld residuals (Grambsch 1995). The analysis showed that the assumption was valid for the treatment group (p = 0.1). Furthermore, the assumption of independent observations may be questioned since some patients with operations in both knees were included (bilateral observations, 9%). However, several studies have found that the effect of including bilateral operations on the results is minor—both for hip prostheses (Lie et al. 2004) and for knee prostheses (Robertsson and Ranstam 2003).

In a subanalysis, a possible effect of a learning curve was investigated by excluding the first 20 operations with CAS at each center. The specific results of each center were investigated and the impact of hospital volume was addressed in a separate subanalysis, by selecting centers with more than 50 CAS cases. Furthermore, a selection of centers performing both operating techniques in the same time period was analyzed.

Secondary outcome measures were investigated using Fisher's exact test for comparison of intraoperative complication rates and the independent-samples t-test for mean operating times.

Statistical significance was set at 0.05. The analyses were done using SPSS software version 17.0 and R (the R Foundation for Statistical Computing).

### Follow-up

The mean follow-up time was 1.4 years in the CAS group and 1.8 years in the CON group.

### Ethics

The Norwegian Arthroplasty Register has permission from the Norwegian Data Inspectorate to collect patient data, based on obtaining written consent from patients (last issued May 24, 2004; reference number 2003/58-3).

## Results

### Overall survivorship (Table 2)

**Figure 2.**
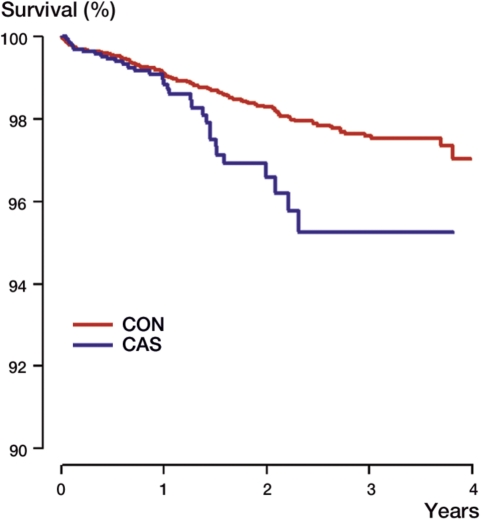
Cox regression survival curves of computer-navigated (CAS) and conventionally operated (CON) primary total knee replacements, without patella resurfacing, reported to the Norwegian Arthroplasty Register 2005–2008.

The CAS group had a higher risk of revision than the CON group (Figure 2). At 1 year, the survival rate was 98.8% (CI: 98.6–99.0) in the CON group and 98.5% (CI: 97.7–99.3) in the CAS group. At 2 years, the survival rates were 97.9% (CI: 97.5–98.3) in the CON group and 96.4% (CI 95.0–97.8) in the CAS group. Cox regression analysis, adjusting for age, sex, prosthesis brand, ASA category, preoperative diagnosis, previous knee surgery, and fixation method, showed a higher relative risk of revision in the CAS group than in the CON group (RR = 1.7, CI: 1.1–2.5; p = 0.02).

**Table 2.** Kaplan-Meier survivorship (KM) and adjusted Cox regression relative risk for conventionally operated (CON) and computer-navigated (CAS) primary total knee replacements without patella resurfacing reported to the Norwegian Arthroplasty Register, 2005–2008

	Revised/ total (%)	MF **^a^** (years)	1 year At risk	1 year KM survival (95% CI)	2 years At risk	2 years KM survival (95% CI)	2005–2008 Cox-adjusted **^b^** relative risk (95% CI)
CON	149/8,214 (1.8%)	1.8	5,776	98.8 (98.6–99.0)	3,520	97.9 (97.5–98.3)	1
CAS	32/1,465 (2.2%)	1.4	757	98.5 (97.7–99.3)	400	96.4 (95.0–97.8)	1.7 (1.1–2.5) **^c^**

**^a^** MF: mean follow-up (reversed KM).

**^b^** Adjusted for sex, age, prosthesis brand, preoperative diagnosis, previous knee surgery, fixation method, and ASA category.

**^c^** P-value = 0.019

### Prosthesis brands (Figure 3)

The mobile-bearing LCS Complete in particular (with all methods of fixation) had a higher risk of revison when inserted with computer-assisted navigation (n = 570) than when inserted by conventional means (n = 2,834) (RR = 2.1, CI: 1.3–3.4; p = 0.004). For the AGC implant (with 80 CAS and 1,072 CON) and the Duracon implant (168 CAS and 443 CON), the relative risks were 1.8 (CI: 0.4–8.0; p = 0.4) and 1.4 (CI: 0.4–5.7; p = 0.6) in favor of the CON group, but there were few revisions and the finding was not statistically significant. A subanalysis of the AGC Anatomic did not show significantly altered results (RR = 1.7, CI: 0.4–7.8; p = 0.5). The Profix (347 CAS and 3,858 CON) appeared to have a lower relative risk (RR = 0.8, CI: 0.1–5.6; p = 0.8) when computer navigated, not statistically significant. Only 1 of the 300 mobile-bearing e.motion knee replacements that was inserted using computer-assisted navigation was revised.

**Figure 3.**
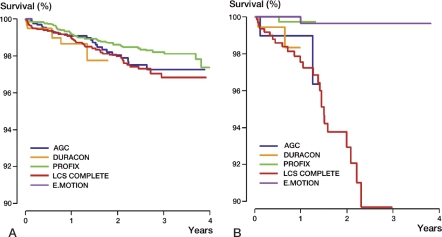
A. Cox regression survival curves of conventionally operated knee replacements (CON) sorted into various prosthesis brands, as reported to the Norwegian Arthoplasty Register 2005–2008. B. Cox regression survival curves of computer-navigated knee replacements (CAS) sorted into various prosthesis brands.

### Fixation method

When only cemented implants were selected (1,087 CAS and 6,794 CON), the relative risk of revision was similar, with a higher risk in the CAS group (RR = 1.8, CI: 1.1–2.8; p = 0.02). Separately, the cemented mobile bearing LCS Complete (421 CAS and 2,521 CON) still had a higher risk of revision in the computer-navigated group (RR = 2.0, CI: 1.3-3.3; p = 0.005).

For the uncemented implants (278 CAS and 111 CON) we found the same tendency, but this was not statistically significant (RR = 1.7, CI: 0.6–4.6; p = 0.3). All revisions in the uncemented group involved the LCS Complete brand. In the hybrid knee replacements (uncemented femur and cemented tibia: 81 CAS and 1,201 CON), the tendency of an inferior outcome for the CAS knees prevailed, but the result was not statistically significant (RR = 3.5, CI: 0.4–31; p = 0.3).

### Intraoperative complications (Table 3)

The frequency of intraoperative complications was similar in both groups. Complications occurring with a frequency of more than 1 in 1,000 cases were included in the analysis. The complications reported were too few to reveal any statistically significant differences, except in the category “anesthesia failure”, which was not reported in the CON group but was reported 3 times in the CAS group.

**Table 3.** Intraoperative complications occurring more frequently than 1 in 1,000

	Instrument failure	Fracture of the tibia	Cement failure	Anesthesia failure	Torniquet failure	Patella tendon rupture/avulsion or ligamentous/tendinous injury
CON	10	15	6	0	8	21
CAS	5	4	3	3	3	4
p-value **^a^**	0.1	0.5	0.1	0.003	0.2	0.8

**^a^** By Fisher's exact test.

### Causes of revision (Table 4)

There was a tendency of more revisions due to deep infection (RR = 1.7, CI: 0.9–3.3; p = 0.1) and loosening of the tibia (RR = 2.1, CI: 0.9–4.9; p = 0.1) in the CAS group, when adjusting for age and sex, but the number of revisions was low and the differences were not statistically significant. Revisions due to malalignment (RR = 0.7, CI: 0.1–5.6; p = 0.7) and instability (RR = 0.6, CI: 0.1–2.4; p = 0.4) were more frequent in the CON group, but this was not statistically significant.

**Table 4.** Total knee replacements reported to the Norwegian Arthroplasty Register, 2005–2008: causes of revision and Cox relative risk (RR) with 95% CI. Computer-navigated TKRs (CAS) and conventionally operated TKRs (CON) are compared. (There may have been more than one cause of revision reported in each case)

	A	B	C	D	E	F	G	H	I	J	K	L
CON, n	8	22	3	4	22	11	36	6	63	6	19	149
CAS, n	0	8	1	0	2	1	13	1	13	0	1	32
RR CAS vs CON	–	2.1	2.4	–	0.6	0.7	1.7	0.9	1.3	–	0.3	
(95%CI)		0.9–4.9	0.2–25		0.1–2.4	0.1–5.6	0.9–3.3	0.1–7.7	0.7–2.5		0.04–2.2	

A Loose femoral component B Loose tibial component C Dislocated patella D Dislocation (not patella) E Instability F Malalignment G Deep infection H Fracture (affecting implant) I Pain J Defect polyethylene insert K Other L Total no. revised

### Learning curve

When the first 20 operations at each center were excluded, the relative risk was 1.8 (CI: 1.1–2.9; p = 0.02) in favor of the CON group, which was similar to the risk without the exclusion.

### Hospital volume and hospital-specific results

When we selected centers performing more than 50 operations with the use of computer navigation, the number of CAS knees was reduced to 1,221, and the statistical power was weaker. There was a tendency of inferior results for the CAS group (RR = 1.4, CI: 0.8–2.6; p = 0.2).

The hospitals performing both techniques were suitable for a direct comparison of the 2 groups. We found the same increased relative risk for the CAS group (RR = 1.6, CI: 1.0–2.6; p = 0.05). When each hospital was checked individually to reveal any difference in survivorship between the CON and CAS groups, the numbers were small and no statistically significant differences were found.

### Operating time

The mean operating time was 107 (SD 33) min in the CAS group and 92 (SD 29) min in the CON group (p < 0.001).

## Discussion

We found that the 2-year risk of revision was higher for the CAS group than for the CON group. Consequently, the effect of improved alignment by computer-assisted navigation on the long-term survivorship must be even greater than previously suggested (Slover et al. 2008) in order to achieve cost-effectiveness with CAS.

### Strengths and limitations

The large number of surgeons and hospitals participating at the national level was a strong point of this study, and resulted in good external validity. The outcome is probably what could be expected by the average surgeon. Previous studies on computer navigation have been done at expert centers with one or a few enthusiastic surgeons, and the main outcome has been alignment. In the present study we concentrated on the clinically important risk of revision and the various reasons for revision.

The number of knee implants that are inserted by computer-assisted navigation in Norway is small, but the number is still sufficient to show statistically significant inferiority of CAS compared to CON, with short-term follow-up. Not all centers performing the operations have been using both methods, so we did a subanalysis to include only centers using both methods over the study period, to allow a more direct comparison. The results of the subanalysis were in favor of CON, but this was not statistically significant. A randomized clinical trial would typically address this problem by comparing the 2 groups directly, with standardized perioperative facilities. On the contrary, a registry study will reflect the results in a general population, with average surgeons and a regular perioperative set-up. The applicability and external validity might be regarded as stronger with a registry study, involving many surgeons from different types of centers and different prostheses, surgical techniques, and experience—and with a higher power to detect differences due to a higher number of patients (Graves 2010).

Considering the fact that 20% of the knee replacements performed in Norway in 2008 were computer-navigated, the inferior short-term results give cause for concern.

### Explanations and mechanisms

1,465 knees is not a large number from a registry point of view, and may have introduced some bias into the results. A learning curve and technical failures related to the computer navigation systems would be expected to negatively affect the survivorship, but exclusion of the first 20 operations at each center did not alter our results. Interestingly, Maniar et al. (2011) found that there was a learning curve with the use of computer navigation, but even patients who were operated during the period of the surgeon's learning curve achieved a better alignment with the navigation technique than with the conventional technique. The outcome, however, was radiographic alignment and not short-term survivorship. The learning curve might involve technical difficulties that compromise the bones, ligaments, soft tissue, and fixation method, which would not be revealed on postoperative radiographs. We regarded 20 patients as representing a reasonable learning curve, but the curve might be steep even after 20 operations, and in some centers these operations are perhaps performed by more than one surgeon with different skills and experience. Insufficient training programs for CAS might lead to a long learning curve with increased complication rates. Perhaps the technical failures related to computer navigation are difficult to avoid—even for experienced surgeons. Our study suggests that there are indeed some technical failures typically related to the computer navigation technique, which may compromise the survivorship. Specifically, the prolonged operating time and the disadvantage of trans-cortical drilling of the tibia and femur to fix the trackers to the bone are of concern (Jung et al. 2007, Bonutti et al. 2008, Li et al. 2008). In our study, there was no evidence of an increased risk of fracture with the use of computer navigation. However, fractures not leading to removal of the implant, or parts of an implant, are not reported to the register unless they occur as an intraoperative complication. Theoretically, the observed prolongation of operating time might increase the risk of revision due to infection, as previously reported for hip replacements (Smabrekke et al. 2004). In our study, however, the prolonged operating time did not give any increased risk of infection. In a separate survival analysis we adjusted for operating time, but the difference in survivorship remained, in favor of CON, indicating that infection was not a major cause of the inferior survivorship in the CAS group.

Our findings also suggest that there are brand-specific problems when matching computer navigation systems and prosthesis brands. The most frequently revised prosthesis brand in the CAS group was the mobile bearing LCS Complete, with survivorship inferior to that of the LCS Complete in the CON group. Thus, our finding might suggest that the LCS Complete is difficult to navigate, perhaps due to the mobile bearing design of the implant or to the brand-specific surgical instruments using gap-measuring technique. The fixed-bearing Profix prosthesis is computer-navigated with the same “open” system (Brainlab) as the LCS Complete, but this combination was not inferior to the conventionally operated Profix knee. Furthermore, the LCS Complete and the e.motion prostheses both have a mobile-bearing polyethylene, but the e.motion—which is closely linked to the “closed” Orthopilot navigation system—had excellent survivorship, with only 1 revision. Thus, the compatibility between computer navigation system and prosthesis brand might be important. “Open” systems are not matched for one prosthesis brand only, but seek to embrace all kinds of implants. “Closed” systems may be more closely matched to the implants, which could be an advantage.

### Comparison with other relevant studies

Our study reveals that unexpected problems may occur when new technology is introduced onto the market. Previous reports have discussed how much of an improvement is needed to render this new procedure cost-effective (Novak et al. 2007, Slover et al. 2008). In contrast, we found that the short-term results on a national basis were inferior with the use of this new technology, thus changing the outlook on whether this technology really is an improvement after all. New kinds of complications may not only neutralize the effect of a better alignment, but might even negatively affect the long-term survivorship.

Some authors have suggested that computer navigation is most helpful in difficult cases with malalignment, fracture sequelae, and abnormal anatomy (Laskin and Beksac 2006). Surgeons may then have selected difficult cases for computer navigation, which could have affected our results. We have adjusted for differences between the two groups, but preoperative malalignment data were not available for analysis. If the most malaligned knees were selected for computer navigation, inferior results might be more likely to occur. This issue should be explored further in randomized clinical trials with long-term follow-up.

Intraoperative complications were similar in both groups, except that “anesthesia failure” was reported 3 times in the computer navigated group and never in the conventionally operated group. This failure might be due to the longer operating time, with loss of spinal anesthesia. Revisions because of malalignment or instability were more frequent, although not statistically significantly so, in the CON group. The computer navigation was primarily introduced to knee replacement surgery to improve the alignment, so this finding was not surprising. The instability might be due to ligamentous imbalance and malaligned implantations (Gorab and Barnett 2002), but again, our numbers were too small to allow us to make any conclusions.

### Possible implications

The introduction of new risk factors with CAS and compatibility problems between CAS systems and specific prosthesis brands could indicate more restricted use of the CAS technology. The results with specific prosthesis brand results were divergent, however, and some brands may have benefited from this new technology while others had inferior results with the use of computer navigation. The explanation for the inferior results with CAS, especially for the LCS Complete, might not only be problems with the computer navigation technology, but they could also be a result of surgical errors introduced along with this new technique. A selection bias from recruiting difficult patients to the CAS group is another explanation that cannot be overlooked, even though we tried to adjust for differences between the groups. Development of faster computer navigation techniques with more user-friendly instruments might improve the short-term results. Long-term registry studies and large randomized clinical trials with a long-term follow-up will be necessary to verify our findings and to explore the failure mechanisms in more detail. However, the rapid evolution of new technology challenges our standards and demands faster evaluation methods. Radiostereometric analysis (RSA) and laboratory tests are some ways of speeding up the evaluation process.

### Conclusion

With the introduction of computer navigation to knee replacement surgery in Norway, the short term risk of revision has increased for the LCS complete implant. Even though the difference is small, improved longevity due to CAS might be unlikely, considering the inferior short term results. The failure mechanisms of these implantations must be explored in greater detail, and we have not been able to draw any conclusions from the present study regarding causation. The success of computer navigation as a surgical instrument may be dependent on the design of the implant; selection bias and the introduction of surgical errors may affect the results. Thus, care has to be taken when introducing new technology into a field of orthopedics where the results are already good.
